# Anti-inflammatory effect of oligostilbenoids from *Vitis heyneana* in LPS-stimulated RAW 264.7 macrophages via suppressing the NF-κB activation

**DOI:** 10.1186/s13065-018-0386-5

**Published:** 2018-02-13

**Authors:** Do Thi Ha, Phung Thanh Long, Tran Thi Hien, Dao Trong Tuan, Nguyen Thi Thuy An, Nguyen Minh Khoi, Ha Van Oanh, Tran Manh Hung

**Affiliations:** 1 Vietnam National Institute of Medicinal Materials, 3B Quangtrung, Hanoi, Vietnam; 2 Department of Experimental Medical Science, Faculty of Medicine, Lund University, BMC D12, 221 84 Lund, Sweden; 3 Thai Binh University of Medicine and Pharmacy, Thai Binh City, Vietnam; 4 Department of Plant and Environmental Sciences, University of Copenhagen, Copenhagen, Denmark; 5 Research Institute of Pharmaceutical Sciences, College of Pharmacy, Kyungpook National University, Daegu, 41556 Republic of Korea; 6 Hanoi University of Pharmacy, 13 Le Thanh Tong, Hoan Kiem district, Hanoi, 100100 Vietnam; 7 Department of Biomedical Sciences, Institute for Research and Executive Education (VNUK), The University of Danang, 41 Le Duan, Haichau district, Danang, 551000 Vietnam

**Keywords:** *Vitis heyneana*, (-)-*Trans*-ε-viniferin, COX-2, PGE2, NO, NF-κB

## Abstract

**Background:**

*Vitis heyneana* is widely distributed in the north of Vietnam, it has been used in Vietnamese traditional medicine as an agent for treatment of arthritis, bronchitis, carbuncles and inflammatory conditions, and menstrual irregularities. However, this plant has not been investigated in phytochemical constituents and biological effects, especially in the anti-inflammatory property.

**Results:**

Bioassay-guided fractionation of the EtOAc soluble fraction from the aerial part of *Vitis heyneana* resulted in the isolation of a series of oligostilbenoids as piceid (**1**), 2-*r*-viniferin (**2**), betulifol A (**3**), vitisinol C (**4**), *(*-*)*-*trans*-*ε*-viniferin (**5**), α-viniferin (**6**), shoreaketon (**7**), amurensin B (**8**), vitisinol B (**9**), and *cis*-vitisin B (**10**). Compound **5** showed the most potent inhibitory activities by suppressing LPS-induced COX-2 expression and PGE2 production. This compound exhibited significantly reduced LPS-induced nitric oxide (NO) release in a dose-dependent manner. These effects are accompanied with the inhibition of transcription factor NF-κB activation.

**Conclusion:**

The results suggested that *trans*-ε-viniferin exerts anti-inflammatory effects via suppression the NF-κB activation in RAW 264.7 cells. 
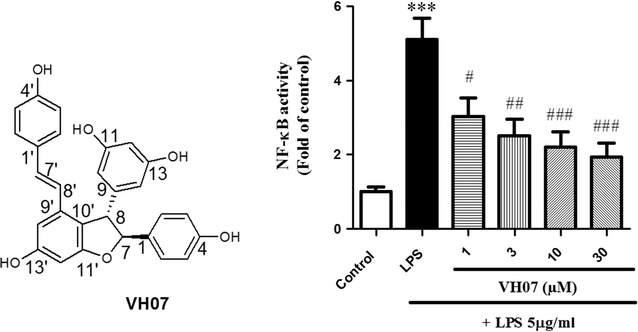

## Background

The genus *Vitis* (family Vitaceae) is in the major group with more than 66 species identified and is distributed throughout the world [1]. In Vietnam, 6 species of *Vitis* genus have been reported until now, including *V. larbusca* L., *V. heyneana* Roem, & Schult., *V. retordii* Roman du Caill. Ex Planch. *V. vinifera* L., *V. balansana* Planch., *V. flexuosa* Thunb [2]. Typical constituents in *Vitis* genus have been reported to be oligomers of resveratrol named stilbenoids [3–7]. To date, over 1000 stilbenoids have been identified in various plant families such as Celastraceae, Cyperaceae, Fabaceae, Iritaceae, Moraceae, Paeoniaceae, and Vitaceae. The Vitaceae family includes about 900 species within 14–17 genera, primarily in tropical climate [8, 9]. Of these genera, only five, the *Ampelopsis, Cissus*, *Cyphostemma*, *Parthenocissus*, and *Vitis* genera reported the presence of stilbenoids. However, chemical constituents of the species in *Vitis* genus have been studied the most. Stilbenoids possessed various pharmacological activities such as antioxidative, anti-inflammatory, and antimicrobial activities, as well as having cardioprotective, hepatoprotective, and neuroprotective effects [10–14]. Although approximately 100 stilbenoid monomers, dimers, and oligomers have been found in all *Vitis* species, research on the chemical compositions and biological activities of *Vitis* genus remains lacking [15]. Our screening anti-inflammatory effect of an ethanol extract of 4 *Vitis* species including *V. heyneana* Roem. & Schult. (VH), *Vitis vinifera* L. (VV); *Vitis balansana* Planch. (VB), and *Vitis labrusca* L. (VL) collected in Vietnam via suppression of LPS-induced COX-2 expression found that 96% of ethanol extract of VH exhibited the most activity [16]. Besides, *V. heyneana* is widely distributed in northern Vietnam, for example in Cao Bang, Lang Son, and Lao Cai provinces. The stems and roots of this species are traditionally used for the treatment of arthritis, bronchitis, carbuncles and inflammatory conditions, and menstrual irregularities in Vietnamese indigenous peoples [17]. So far, few studies have been done to investigate the chemical constituents of VH. Currently, only a few studies have referred to the presence of stilbenoids in VH [17–19]. However, evaluation of anti-inflammatory activities of the VH species have not been studied yet. This study reports the anti-inflammatory effects of VH extracts and its isolated oligostilbenoids via suppressing LPS-induced COX-2 expression, PGE2 and NO productions, and NF-κB activation in RAW 264.7 macrophages.

## Results and discussion

### Screening inhibitory activities

**Fig. 1:**
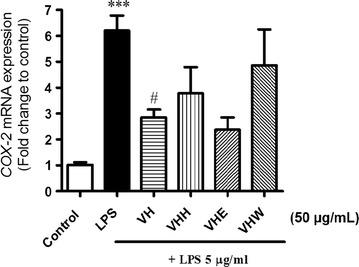
COX-2 expression effects of *V. heyneana* extract (**VH**) and its fractions (VHH: *n*-hexane, VHE: ethyl acetate, VHW: water fraction; all 50 µg/mL). Eighteen hours after treating cells with LPS (5 µg/mL) with or without fractions in RAW 264.7 cells. Samples were harvested and lysated for COX-2 mRNA level by qPCR. Relative changes in the COX-2 mRNA expression (*significant as compared to control, *, *p* < 0.05; **, *p* < 0.01; ***, p < 0.001; ^#^significant as compared to LPS group, n = 4–5)

To study the cytotoxic effects of extract and its fractions on cell viability, the RAW 264.7 cells were incubated and treated a concentration of all materials (50 µg/mL). The results showed that all the extract and fractions that induced cell toxicities were negligible at the above concentrations [16]. In order to examine candidate extract/fractions inhibiting COX-2 in RAW 264.7 cells, ethanol *V. heyneana* extract (VH) and its fractions (*n*-hexane-VHH, ethyl acetate-VHE and water-VHW) were tested on COX-2 mRNA expression level by qPCR. As shown in Fig. 1, the inhibitory effect of COX-2 mRNA was mostly potently suppressed by VHE and VH.

### Structure identification of the isolated compounds

**Fig. 2:**
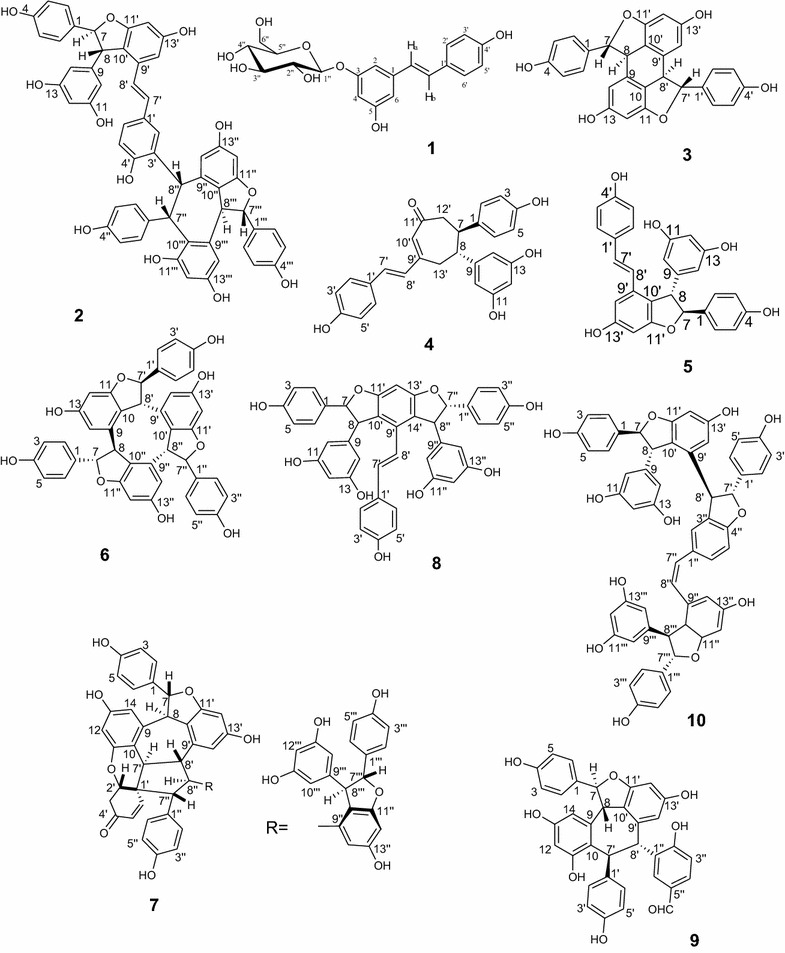
Chemical structures of oligostilbenoids from *V. heyneana* (**1**–**10**)

To investigate the active components from the potential fraction, several chromatographic techniques were applied and ten compounds were obtained after purification. On the basis of NMR spectroscopic analysis, and in comparison with the previous studies, the chemical structures of these compounds were identified as piceid (**1**), 2-*r*-viniferin (**2**), betulifol A (**3**), vitisinol C (**4**), (-)-*trans*-ε-viniferin (**5**), α-viniferin (**6**), shoreaketon (**7**), amurensin B (**8**), vitisinol B (**9**), and *cis*-vitisin B (**10**) (Fig. 2) [17, 20–26].

### Screening inhibitory activities of oligostilbenoids by suppressing LPS-induced COX-2 expression and PGE2 production in RAW 264.7 macrophages

**Fig. 3:**
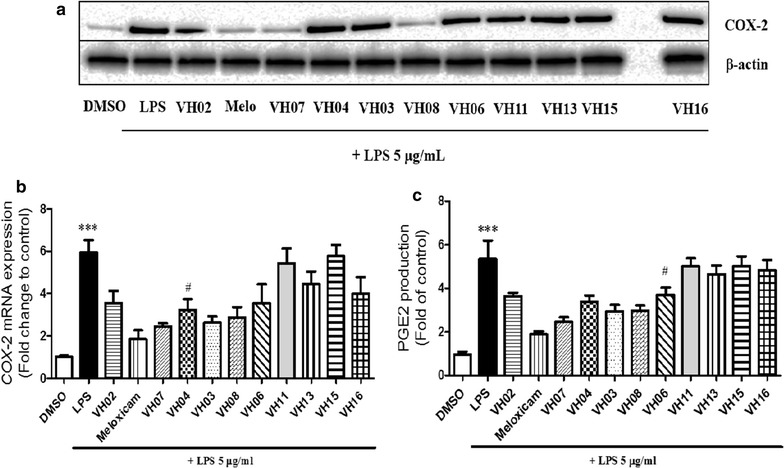
Comparison of COX-2 protein expression effects of compounds from *V. heyneana.* 18 h after treating cells with LPS (5 μg/mL) with or without compounds in RAW 264.7 cells, samples were harvested and lysated to immunoblottings with COX-2 and β-actin antibodies. **b** COX-2 mRNA level by qCPR (***significant as compared to control, *p < 0.05; ^#^significant as compared to LPS group, n = 5. **c** Comparison of PGE2 production effects of all compounds (10 M). RAW 264.7 cells were incubated with 5 μg/mL LPS for 18 h with or without compounds and amount of PGE2 in medium was determined using PGE2-specific ELISA assays (***significant as compared to control, *p < 0.05; ^#^significant as compared to LPS group, n = 5. Codes: VH02 compound 1; VH03 - 2; VH04 - 3; VH06 - 4; VH07 - 5; VH08 - 6; VH11 - 7; VH13 - 8; VH15 - 9; and VH16 - 10.

The effects of all isolated compounds at concentration of 10 µM, and a reference agent, meloxicam (20 µM) were further examined on LPS-induced COX-2 protein, mRNA expression and LPS-induced PGE2 production in RAW 246.7 cells by reported method with slight modification. As the results in Fig. 3, western blotting and real time-PCR assays revealed that, among the active metabolites, *trans*-ε-viniferin (**5**; 10 µM) potently suppressed LPS-induced COX-2 expression in RAW 246.7 cells (Fig. 3a, b). Furthermore, ELISA assay confirmed that **5** most inhibited the level of LPS-induced PGE2 production in RAW 264.7 cells (Fig. 3c).

### Effects of 5 on COX-2, iNOS expression and PGE2 production in RAW 264.7 macrophages

**Fig. 4:**
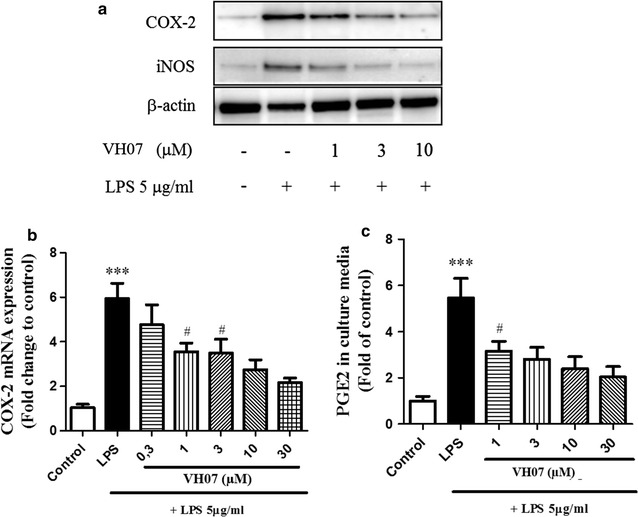
Effect of **5** on COX-2 and iNOS expression in dose-dependent manner (1–30 µM). Raw264.7 cells were treated with 5 μg/mL LPS for 18 h with or without VH07 and then harvested and lysated to immunoblottings with COX-2, iNOS and β-actin antibodies. **b** Effect of **5** on LPS-induced COX-2 was analyzed by qPCR (***significant as compared to control, *p < 0.05; ^#^significant as compared to LPS group, n = 5). **c** Effect of **5** on PGE2 production. RAW 264.7 cells were incubated with 5 μg/mL LPS for 18 h with or without **5** and amounts of PGE2 in medium was determined using PGE2-specific ELISA assays. (***significant as compared to control, *p < 0.05; ^#^significant as compared to LPS group, n = 4)

Considering that the **5** was the most active metabolite, the effects of this compound was investigated on LPS-induced PGE2 production in RAW 264.7 cells using ELISA method with slight modification. As the results in Fig. 4a show, stimulation of cells resulted in the increase of PGE2 production compared with unstimulated vehicle cells. In the administration of **5** (1–30 µM), the PGE2 productions were remarkably reduced in a concentration dependent manner (*p* < 0.05). We further investigated whether the inhibition effect by the same range of concentration of **5** was related to the gene expression, especially in quantitation protein and mRNA level of COX-2 by performing western blotting and real-time PCR. As shown in Fig. 4b and c, COX-2 levels were decreased by both of protein amount and mRNA levels. These results were also confirmed by dose dependent manner.

### Effect of 5 on NO productions and NF-κB activation

**Fig. 5:**
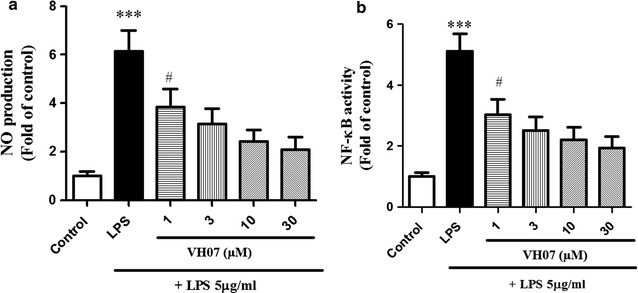
Compound **5** (VH07) reduced LPS-induced NO production and NF-κB activity in RAW 264.7 macrophages in a dose-dependent manner. ***significant as compared to control, *p < 0.05; ^#^significant as compared to LPS group, n = 4

As shown in Fig. 5a, **5** (Code VH07, 1–30 µM) significantly inhibited LPS-induced NO production in a dose dependent manner. Especially, at the highest concentration (30 µM), this compound could decrease the amount of NO production more than 2-fold compared to unstimulated vehicle. Since NF-κB was identified as an important transcription factor that controls several pro-inflammatory mediations, we investigated the NF-κB transcription activity by performing luciferase reporter gene assay and the results are shown in Fig. 5b. Compound **5** (1–30 µg/mL) dose dependent reduced LPS-induced NF-κB transitivity (*p* < 0.05).

## Discussion

*Vitis* sp. is widely distributed and has been used as the raw material for juice and wine all over the world. For the pharmaceutical application, it has been reported that the roots, stems and leaves possessed anti-inflammatory, antioxidants, and anti-tumour activities, and contains a number of stilbenoid and resveratrol oligomers. Previously, resveratrol (3,4′,5-trihydroxystilbene) isolated mostly from *Vitis* sp. as a main metabolite with high concentration, was shown to play an important role in human health with extremely extensive bioactivities such as anti-bacterial, anti-thrombotic, anti-oxidation, anti-inflammatory, reduce hypertension, and especially anti-cancer [9–13]. In addition, several studies focused on resveratrol oligomers that are characterized by the polymerization of several resveratrol units [9–13]. In this study, we demonstrated the potential involvement of the NF-κB pathway in the anti-inflammation of metabolites from *V. heyneana*. Among the active components, **5** was found to be the most anti-inflammatory activity in suppression of NO, PGE2, and COX-2 production in LPS-stimulated RAW 264.7 macrophage cells. We also found that, this oligostilbenoid inhibited LPS-induced NF-κB activation.

RAW 264.7 cells are the murine macrophage cells line that plays an important testable model for anti-inflammatory agent nowadays. When the macrophage cells were activated by LPS, a number of cytokines were released and recordable. Among them, NO is an inflammation mediator, in which, NO produced by iNOS coursed toxicity to cells and directly concern to the pathogenesis of inflammation process. COX-1 and COX-2 are the isozymes that convert arachidonic acid to prostaglandin, however, COX-2 responses mainly to produce a huge amount of PGEs in macrophage cells. Inhibition of the above productions may be the effective method for the treatment of several types of inflammation. Of our experiments, this is the first report confirming that oligostibene from *V. heyneana* suppresses the LPS-induced inflammatory response by activating NF-κB in vitro. (-)-*Trans*-ε-viniferin was first isolated from *V. vinifera* and classified as a model for its bio-synthesis from resveratrol. Similar to resveratrol, this dimer was shown to have several biological properties such as anti-oxidant, antidepressant, and anti-adipogenesis [27–30]. This compound showed cytotoxicity in several cancer cell lines as C6, Hep G2, HeLa, and MCF-7n and exerted the anti-proliferative and pro-apoptotic effect in U266, RPMI8226, Jurkat, K562 and U937 and other cancer cell lines [4, 31]. (-)-*Trans*-ε-viniferin and its derivative compounds slightly reduced cell proliferation on human adenocarcinoma colon cells and could constitute new putative anti-cancer agents on colon carcinoma [32]. (-)-*Trans*-ε-viniferin significantly attenuated mutant Htt-induced depletion of SIRT3 and protected cells from mutant Htt. This compound also decreased levels of reactive oxygen species and prevented loss of mitochondrial membrane potential in cells expressing mutant Htt [33]. The other form, α-viniferin, also down-regulated the LPS-induced expression of pro-inflammatory genes such as iNOS and COX-2 by suppressing the activity of NF-κB via dephosphorylation of Akt/PI3K. This compound suppressed NO and PGE2 production in the late stage of inflammation through induction of heme oxygenase-1 (HO-1), and the expression of pro-inflammatory genes iNOS and COX-2 in the early stage of inflammation by inhibiting the Akt/PI3K-dependent NF-κB activation in BV2 microglial cells [34]. The *V. thunbergii* extract that was rich in (-)-*trans*-ɛ-viniferin significantly inhibited PGE2 production in LPS-induced PHCs cells without exhibiting significant cytotoxicity [35].

Even though (-)-*trans*-ɛ-viniferin and its isomers have been shown to have several anti-inflammatory effects, the molecular mechanism underlying the anti-inflammatory in LPS-induced RAW 264.7 has not been completely elucidated thus far. The results of our research indicate that among the active components, treating the RAW 264.7 macrophage cells with several concentrations of (-)-*trans*-ɛ-viniferin (**5**) could inhibit LPS-induced NO, PGE2, iNOS, COX-2 productions in a dose dependent manner. With the highest concentration at 30 µM, this oligostibene significantly reduced the above productions more than 50% as compared to the LPS-treated cell alone.

## Methods

### Plant materials

The aerial parts of *V. heyneana* were collected from Lao Cai province (north of Vietnam) in September 2016 and botanically identified by Assoc. Prof. Dr. Nguyen The Cuong, Institute of Ecology and Biological Resources. A voucher specimen (TL07) has been deposited at the Herbarium of IEBR and Department of Phytochemistry, Hanoi, Vietnam.

### General experiment procedures

Melting points were determined on an Electrothermal apparatus. Optical rotations were measured on a JASCO V-550 UV/Vis spectrometer (Tokyo, Japan). The NMR [^1^H (500 MHz), ^13^C (125 MHz)] experiments were performed on a Bruker Advance 500 spectrometer (United State). Chemical shift was reported in ppm downfield from TMS, with *J* in Hz. Mass spectra were obtained with an AGILENT 1200 series LC-MSD Ion Trap (United State). Analytical TLC was performed on Kieselgel 60 F_254_ (Merck) plates (silica gel, 0.25 mm layer thickness) and RP-18 F_254_ (Merck) plates (0.25 mm layer thickness). UV spots were visualized using ultraviolet irradiation (at 254–365 nm) and by spraying with 10% H_2_SO_4_, followed by heating with a heat gun. Column chromatography was performed on silica gel (70–230 and 230–400 mesh, Merck), YMC RP-18 resin (30–50 μm, Fuji Silysia Chemical Ltd.), and Sephadex™ LH-20 columns (Amersham Biosciences, Uppsala, Sweden). Dulbecco’s modified Eagle’s medium (DMEM), trypsin and fetal bovine serum (FBS) were purchased from Gibco BRL (Grand Island, NY). COX-2 (1:1000, Cat: 610204) was obtained from BD Biosciences. Secondary mouse or rabbit HRP-conjugated antibodies (1:5000 or 1:10,000, Cell Signaling, #7074, #7076). β-actin (Cat: A5316), LPS (Cat: L4391), meloxicam (cat: M3935) and MTT (cat: M2128) were purchased from Sigma-Aldrich.

### Cell culture

RAW 264.7 cells were purchased from the American Type Culture Collection (ATCC, Rockville, MD). Cells were cultured and growth in DMEM (Gibco) medium containing 10% fetal Bovine Serum (FBS) (Gibco), 100 units/mL penicillin, and 100 μg/mL streptomycin at 37 °C in 5% CO_2_-95% air. Penicillin and streptomycin were obtained from Biochrom.

### Western blot analysis

RAW 264.7 cells were seeded in 6 well-plate and treated with extractions or compounds from *V. heyneana.* Cells were collected and washed with cold phosphate-buffered saline (PBS). The collected cells were lysed on ice for 30 min in 100 μL lysis buffer [120 mM NaCl, 40 mM Tris (pH 8), 0.1% NP40 (Nonidet P-40)] and centrifuged at 12,000 rpm for 30 min. BCA protein assay kit (Pierce, Rockford, IL) was used to measure protein concentrations as described in a previous report [36]. Finally, bands were detected by ECL kit (Pierce West Femto), and images were acquired using the Odyssey Fc Imager (LI-COR Biosciences).

### Quantitative real-time PCR (qRT-PCR)

Total RNA was isolated using Trizol (Takara, Japan) according to the manufacturer’s instructions. COX-2 mRNA expression was analyzed by real-time qPCR (StepOnePlus qPCR cycler, Applied Biosystems) using QuantiFast SYBR Green RT-PCR Kit Qiagen, (#204156) and primers (Qiagen): COX-2 (Ptgs2) (Cat: QT00165347), Mm_GADPH (Cat: QT01658692).

### Measurement of nitrite

RAW 264.7 cells (5 × 10^5^ cells) were pre-incubated at 37 °C for 12 h in serum-free medium and NO production was monitored by measuring nitrite levels in culture media using Griess reagent (1% sulfanilamide, 0.1% *N*-1-naphthylenediamine dihydrochloride, and 2.5% phosphoric acid). Absorbance was measured at 540 nm after incubating for 10 min.

### Reporter gene assay

One µg of the plasmid NF-κB or 50 ng of pRL Renilla was transfected into the cells using LipofectAMINE2000 (Invitrogen Corp., Carlsbad, CA) using the Dual-Luciferase Reporter Assay Systems (Promega, Madison, WI, USA). After 6 h, the transfection medium was replaced with the DMEM without serum and the cells were further incubated for 18 h. The firefly and hRenilla luciferase activity was detected using a multilaber counter. Ratio of activity was determined by normalizing the promoter-driven luciferase activity versus hRenilla luciferase.

### Enzyme-linked immunosorbent assay (ELISA)

Prostaglandin E2 (PGE2) concentrations in DMEM media were measured by ELISA kit (Cayman Chemical, Ann Arbor, MI) according to the manufacturer’s protocols.

### Extraction and isolation

The aerial part of *V. heyneana* (5.0 kg) was extracted with EtOH 96% (3 h × 3L) at 50 °C. The combined extracts were filtered and evaporated under pressure to give green residue (235.5 g) which was suspended in water and partitioned with organic solvent to get *n*-hexane (26.05 g), EtOAc (129.67 g), and aqueous extract (50.4 g), successfully. The EtOAc extract (34.0 g) was initially chromatographed on a silica gel column (63–200 µM particle size, Merck) eluting with a stepwise gradient of methylene chloride (MC)-MeOH (from 100:1 to 1:100) to yield seven fractions (VHE1-VHE7). Fraction VHE4 (1.5 g) was fractionated by normal-phase silica gel CC (40–63 µM particle size, Merck) eluted with MC-MeOH (20:1) to obtain compound **3** (13 mg) and five smaller fractions (VHE4.1-VHE4.5). Fraction VHE4.3 Fraction VHE4.4 (500 mg) was subjected to Sephadex eluted with a mixture of MeOH-H_2_O (2:1) to give **4** (23 mg) and **5** (40 mg). Fraction VHE5 (3.5 g) was isolated by silica gel CC with elution mixture of MC-MeOH (15:1) to yield ten sub-fractions (VHE5.1-VHE5.10). Compound **6** (25 mg) was purified from fraction VHE5.3 (400 mg) by using Sephadex LH-20 eluting with MeOH-H_2_O (5:1). Fraction VHE5.4 (1.1 g) was applied to RP-C18 gel CC using a gradient mixture of MeOH-H_2_O (from 1:2 to 2:1) to obtain **7** (25 mg), **8** (22 mg), and **9** (13 mg) and eight fractions (VHE5.4.1-VHE5.4.8). Compound **10** (14 mg) was purified from fraction VHE5.4.5 (300 mg) by using MCI gel CC eluting with MeOH-H_2_O (3:2). Fraction VHE6 (1.3 g) was separated on Sephadex gel CC eluting with MeOH-H_2_O (3:2) to yield **1** (12 mg), and **2** (25 mg). Structural identification of the isolated compounds **1**–**10** have been previously published by our group [37, 38].

(-)-*Trans*-ε-viniferin (**5**): Grey-brown solid; mp150–152 °C, soluble in ethanol, methanol, and acetone; $\left[ \alpha \right]_{\text{D}}^{25}$ -47.0 (*c* = 0.5, MeOH); R_*f*_ = 0,64 (TLC, silica gel, dichlomethane-methanol 7:1, *v/v*). ^1^H-NMR (500 MHz, acetone-*d*_6_), *δ*_H_ (ppm): 7.20 (2H, *d*, *J* = 8.5 Hz, H-2, 6), 7.17 (2H, *d*, *J* = 8.5 Hz, H-2′,6′), 6.90 (1H, *d*, *J* = 16.5, H-7′), 6.83 (2H, *d, J* = 8.5 Hz, H-3, 5), 6.73 (2H, *d, J* = 9.0 Hz, H-3′,5′), 6.72 (1H, brs, H-14^ʹ^), 6.71 (1H, *d, J* = 16.5 Hz, H-8′), 6.33 (1H, *d, J* = 2,0 Hz, H-12′), 6.24 (3H, s, H-10, 12, 14), 5.42 (1H, *d, J* = 5.5 Hz, H-7), 4.47 (1H, *d*, *J* = 5.5 Hz, H-8); ^13^C-NMR (125 MHz, acetone-*d*_6_), *δ*_C_ (ppm): 162.5 (C-11′), 159.9 (C-11, 13), 159.6 (C-13′), 158.2 (C-4, 4′), 147.4 (C-9), 136.4 (C-9′), 133.9 (C-1), 130.1 (C-7′), 129.9 (C-1′), 128.7 (C-2′, 6′), 127.9 (C-2, 6), 123.5 (C-8′), 119.8 (C-10′), 116.3 (C-3′,5′), 116.2 (C-3, 5), 107.0 (C-10, 14)), 104.2 (C-14′), 102.1 (C-12), 96.8 (C-12′), 93.9 (C-7), 57.1 (C-8). ESI–MS *m/z*: 477 [M + Na]^+^.

### Statistics

Values are presented as mean ± SE unless otherwise stated. *P*-values were calculated by Student’s *t* test or one-way analysis of variance followed by Bonferroni post hoc testing using GraphPad Prism 5 (GraphPad Software Inc.). *P* < 0.05 was considered statistically significant. Data are expressed as mean ± SEM. *, *p* < 0.05; **, *p* < 0.01; ***, *p* < 0.001.

## Conclusion

This the first time that a Vietnamese *V. heyneana* extract and its phytochemical constituents have been reported to possess an anti-inflammatory activity. The results demonstrated that one of the most active compounds, (-)-*trans*-ɛ-viniferin, decreased NO, PGE2, iNOS, and COX-2 productions in RAW 264.7 macrophage cells after LPS stimulation. This anti-inflammatory activity may mediate by the NF-κB activation mechanism in the RAW 264.7 cells. However, to imply that this *Vitis* sp. and its components may be useful in the prevention or treatment of inflammatory diseases may be premature as further studies and confirmation of these results are required.
